# Au Nanoparticles Functionalized Covalent-Organic-Framework-Based Electrochemical Sensor for Sensitive Detection of Ractopamine

**DOI:** 10.3390/foods12040842

**Published:** 2023-02-16

**Authors:** Shuying Yang, Ruixi Yang, Jingyi He, Yu Zhang, Yahong Yuan, Tianli Yue, Qinglin Sheng

**Affiliations:** Laboratory of Nutritional and Healthy Food-Individuation Manufacturing Engineering, Research Center of Food Safety Risk Assessment and Control, College of Food Science and Technology, Northwest University, Xi’an 710069, China

**Keywords:** food safety, food additive sensing, ractopamine, electrochemical detection, covalent organic frameworks, nanocomposites, Au nanoparticles

## Abstract

Ractopamine, as a feed additive, has attracted much attention due to its excessive use, leading to the damage of the human nervous system and physiological function. Therefore, it is of great practical significance to establish a rapid and effective method for the detection of ractopamine in food. Electrochemical sensors served as a promising technique for efficiently sensing food contaminants due to their low cost, sensitive response and simple operation. In this study, an electrochemical sensor for ractopamine detection based on Au nanoparticles functionalized covalent organic frameworks (AuNPs@COFs) was constructed. The AuNPs@COF nanocomposite was synthesized by in situ reduction and was characterized by FTIR spectroscopy, transmission electron microscope and electrochemical methods. The electrochemical sensing performance of AuNPs@COF-modified glassy carbon electrode for ractopamine was investigated using the electrochemical method. The proposed sensor exhibited excellent sensing abilities towards ractopamine and was used for the detection of ractopamine in meat samples. The results showed that this method has high sensitivity and good reliability for the detection of ractopamine. The linear range was 1.2–1600 μmol/L, and the limit of detection (LOD) was 0.12 μmol/L. It is expected that the proposed AuNPs@COF nanocomposites hold great promise for food safety sensing and should be extended for application in other related fields.

## 1. Introduction

In recent years, food safety has become a global concern due to the popularity of healthy diets. Due to the diversity of food types, the unknown source of pollution and the accuracy and timeliness of test results, there are also some regulatory blind spots in the field of food supervision. Therefore, the development of rapid, efficient and multiple food pollutant detection technologies has always been a difficult and hot spot in food safety research. In order to improve the taste of the product, some additives are added to food and livestock products. Among them, as an important neurotransmitter in the human central nervous system, ractopamine [1,2] is added to the feed to make livestock and poultry muscle grow faster, increasing lean meat content. However, if it is not used in a standardized manner, it will cause irreversible damage to the human nervous system and physiological function. Proclamation No. 176 issued by the Ministry of Agriculture in 2010 in China banned the use of ractopamine hydrochloride in feed and animal drinking water. The current Chinese national standard GB/T 20189-2006 is “Determination of Ractopamine in Animal Feed by High Performance Liquid Chromatographic Method”, which stipulated that the addition of ractopamine hydrochloride in feed is prohibited. This standard also influences China’s imports and exports of the pork trade worldwide [3]. Therefore, it is of great practical value to construct an analysis and detection technology for ractopamine in food samples.

There have been many analytical methods for the determination of ractopamine at present, such as high-performance liquid chromatography [4], liquid chromatography–tandem mass spectrometry [5], gas chromatography–mass spectrometry [6], enzyme-linked immunosorbent assay [7]. However, the above methods are costly, laborious and lack reproducibility. Electrochemical sensing has advantages of low cost, sensitive response and simple operation [8,9], causing serious concern in the fields of pharmacy, clinical diagnosis, environmental monitoring, food safety, etc. [10,11,12]. Up to now, various new nanomaterials have been used to prepare valuable new electrochemical ractopamine sensors. Electrochemical sensors are considered to be the most effective method for detecting ractopamine [13]. The use of modified electrodes to detect ractopamine is a constant research area in food, clinical and health areas. For examples, Mahmoud et al. reported an electrochemical biodevice developed for the highly selective detection of ractopamine in feed under a double recognition strategy of the aptamer (Apt) and molecular imprinting polymer (MIP) on a glassy carbon electrode (GCE). The detection limit was 330 aM and the linear range was 1 fM to 1.90 µM. In addition, the senor was also applied to urine samples [14]. Cao L.P. et al. developed a kind of electrochemical biosensor using a biochar-supported Cu^2+^/Cu^+^ composite as an electrochemical sensing interface for detecting ractopamine, which was beneficial to the accumulation and electrochemical sensing of targets. The good linear relationship was in the range of 0.1–1.75 μM, and the detection limit was 0.041 μM [15]. Murugan K et al. prepared a disposable electrochemical sensor using titanium nanoparticles (Ti NPs)-anchored functionalized multi-walled carbon nanotube (Ti@f-MWCNTs) composite as electrochemical sensing interface for the detection of ractopamine. The sensor had a wide detection range (0.01–185 μM) for ractopamine and a low detection limit of 0.0038 µM [16]. Janus particles can also be used as a sustainable electrode modifier material for the electrochemical monitoring of the ractopamine [17]. However, most electrode materials have various defects in terms of a low effective electroactive surface area and lack of predictable structure. Therefore, seeking a new type of composite nanomaterials with controllable structure, high specific surface area and good catalytic performance has become the main trend to improve the electrochemical performance.

Since first being introduced by Yaghi et al. in 2005, COFs have been prepared in various shapes and multi-dimensional frameworks (2D-COFs, 3D-COFs) [18,19,20]. COFs have been widely used in the field of electrochemical sensing due to their excellent properties [21,22]. Compared with MOF materials, COFs have the advantages of low cost, easy synthesis, low toxicity and better biocompatibility [23]. On the one hand, the ordered microporous or mesoporous structure of COFs can effectively limit the agglomeration and growth of metal nanoparticles during the reduction process, thereby stabilizing the ultrafine nanostructures and regulating the electronic structure and stereochemistry during the catalytic process. On the other hand, COFs have a large specific surface area, providing a large active surface for the loading of electroactive molecules. In addition, their high acid and alkali resistance and excellent biocompatibility can further improve the stability of electrochemical sensors [24]. Tian et al. [25] successfully prepared a novel composite material using COF nanosheets as a matrix to support the growth of gold nanoparticles, after which the synthesized hybrid nanosheets were used as a new platform for multiplexed detection of hepatitis A virus DNA and hepatitis B virus DNA. Moreover, the proposed sensor was also applied for K^+^ sensing and imaging in living cells. Xin et al. [26] used COFs to modify carbon paste electrodes for electrochemical detection of hydroquinone and catechol. The results show that COF-modified carbon material is a promising candidate material for electrochemical detection of phenolic compounds. Functionalization of the synthesized COFs nanomaterials can further improve the selectivity, specificity and sensitivity of functional nanomaterials to target trace substances [27]. Among them, metal nanoparticles, especially AuNPs promote electron transfer at the electrochemical sensing interface as an efficient catalyst based on AuNPs@COFs.

In this work, nanocomposites (AuNPs@COFs) were successfully prepared by in situ reduction method at room temperature using COFs materials as precursors. The prepared nanocomposites were characterized by TEM, FTIR and Zeta potential analyzers to analyze their morphology, element composition and surface charge. The electrochemical sensing performance of AuNPs@COF-modified glassy carbon electrode for ractopamine was investigated by electrochemical method. Based on AuNPs@COF nanocomposites, a ractopamine electrochemical sensor was constructed to detect ractopamine in actual food samples.

## 2. Materials and Methods

### 2.1. Chemicals and Reagents

2,5-Divinyl-1,4-phthalaldehyde (DVA, 98%) and 1,3,5-tris (4-aminophenyl) benzene (TAPB, >93.8%) were purchased from Jilin Chinese Academy of Sciences-Yanshen Technology Co., Ltd (Jilin, China). Acetonitrile (C_2_H_3_N, >99%), acetic acid (AcOH, 99.5%), tetrahydrofuran (THF, 99.9%), sodium citrate (C_6_H_5_Na_3_O_7_, ≥99%), dopamine (≥99.8%), isoxsuprine (>99%), ritodrine (99.5%), fenoterol (99%) and ethanol (C_2_H_6_O, ≥99.7%) were purchased from Shanghai Aladdin Biochemical Technology Co., Ltd. (Shanghai, China) Chloroauric acid (HAuCl_4_) was purchased from Shanghai Sixin Biotechnology Co., Ltd. (Shanghai, China) Ascorbic acid (AA, ≥99.7%) was purchased from Tianjin Tianli Chemical Reagent Co., Ltd. (Tianjin, China) Uric acid (UA, ≥99%) and glucose (Glu) were purchased from Tianjin Kermel Chemical Reagent Co., Ltd. (Tianjin, China) Ractopamine was purchased from Energy Chemical Technology Co., Ltd. (Shanghai, China).

### 2.2. Apparatus and Characterization

A G2 F20 S-TWIIN high-resolution transmission electron microscope was used in this experiment (FEI Tennessee, USA). A Fourier transform infrared spectrometer (FT-IR) was recorded using a Vertrx 70 FT-IR spectrometer (Broker, Germany). The Zeta potential were measured using a Zeta potential analyzer (Malvern Panalytical, UK). All electrochemical and current-voltage measurements were performed on a CHI660D electrochemical analyzer (Shanghai Chenhua Instrument Co., Ltd., Shanghai, China).

### 2.3. Preparation of COFs Composite Nanomaterials

Spherical COFs nanomaterials were prepared by the following steps [28]:

Preparation and optimization of COFs: The uniform morphology spherical COFs nanomaterials were synthesized under the action of catalyst between amino groups and aldehyde groups by polycondensation at room temperature and then simply cleaned. Firstly, 0.014 g of TAPB and 0.011 g of DVA were put together in a 10 mL centrifuge tube. Then, 5 mL acetonitrile was added to the centrifuge tube and was treated with ultrasound for 2 min. Then, 0.2, 0.4, 0.6, 0.8, 1.0, 1.2, 1.4, 1.6 mL, 12 mol/L glacial acetic acid were dripped, respectively. Subsequently, the mixture was shaken vigorously for 15 s and allowed to stand at room temperature for 72 h. The obtained yellow precipitate was centrifuged at 9600 rpm, and then washed several times with anhydrous THF and ethanol until the lotion was clear. Finally, the resulting sample was dried at a high vacuum of 60 °C for about 24 h.

Preparation of AuNPs@COFs nanocomposites: AuNPs@COFs nanocomposites were prepared by in situ growth method at room temperature [29]. The 7 mg spherical COFs with the best morphology and size were transferred to 10 mL of 1 mmol/L HAuCl_4_ solution. The mixture was then stirred on a magnetic stirrer for 5 min. Subsequently, the sodium citrate solution was slowly added. After 30 min of reaction at room temperature, the mixture was centrifuged at 6000 rpm for 5 min. To collect the pure composite material, the precipitate was washed four times with ultrapure water. Finally, the obtained dark-brown product was dried at 60 °C in vacuum for 16 h.

### 2.4. Construction of AuNPs@COFs Nanocomposites Electrochemical Sensor

**Figure 1:**
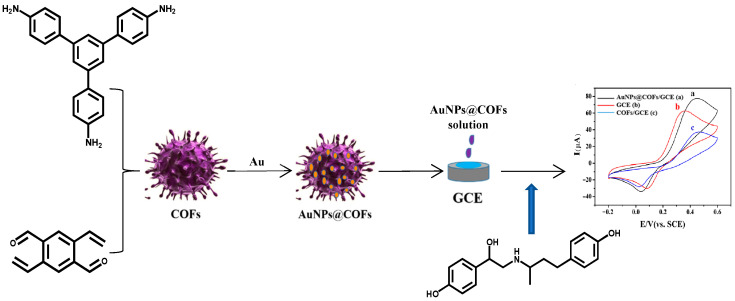
Preparation and sensing principle diagram of AuNPs@COFs nanocmposites.

Before modification, the bare glassy carbon electrode (GCE) with a diameter of 3 mm was ground with 0.05 μm Al_2_O_3_ powder, washed with ultrapure water, and then ultrasonically cleaned with a mixture of ethanol and ultrapure water for 2 min. After drying, the surface of the bare GCE remained as smooth as a mirror. 1 mg AuNPs@COFs nanocomposites were uniformly dispersed in 1 mL 0.5% chitosan solution to obtain a mixed solution with a concentration of 1 mg/mL. Finally, 7 μL of the mixed solution was dropped on the surface of the prepared GCE and dried naturally at room temperature to obtain the modified electrode AuNPs@COFs/chitosan/GCE. At the same time, COFs/chitosan/GCE were prepared using the same method. Figure 1 shows the electrode modification and subsequent detection process.

### 2.5. Pretreatment of Actual Samples

Pork and chicken were purchased at the local market. The two samples were processed in the following steps [30]: 2.0 g of pork and chicken samples were weighed, added in acetonitrile, and were twisted into a uniform paste. Then, the homogeneous solution was centrifuged at 6000 rpm for 9 min, and the supernatant was collected. Next, the precipitate was dissolved in acetonitrile, and the above steps were repeated again. Secondly, the supernatant was collected. The twice-collected supernatant was mixed and filtered through a membrane with a pore size of 0.2 μm. The collected liquid was evaporated to 2 mL. Finally, the resulting extract was precisely added into ultrapure water to 5 mL and stored in a refrigerator at 4 °C.

## 3. Results and Discussion

### 3.1. Characterization of COFs Nanomaterials and AuNPs@COFs Nanocomposites

**Figure 2:**
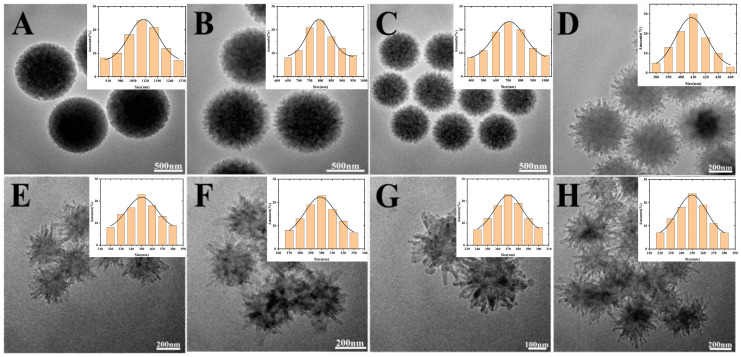
TEM images and particle diameter distribution of uniform spherical COFs nanomaterials with different sizes prepared in 0.2, 0.4, 0.6, 0.8, 1.0, 1.2, 1.4 and 1.6 mL HAc solution: (**A**) 0.2 mL, 1100 nm; (**B**) 0.4 mL, 800 nm; (**C**) 0.6 mL, 700 nm; (**D**) 0.8 mL, 400 nm; (**E**) 1 mL, 350 nm; (**F**) 1.2 mL, 300 nm; (**G**) 1.4 mL. 270 nm; (**H**) 1.6 mL, 250 nm.

As shown in Figure 2, the prepared COFs nanomaterials have a certain uniformity and dispersion. When the concentration of acetic acid (HAc) remained unchanged at 12 mol/L, the average diameter of spherical COFs increased from 250 nm to 1.1 μm while the amount of HAc increased from 0.2 mL to 1.6 mL. The effect of HAc content on the morphology of COFs was also investigated. In Figure 2A–H, the amounts of HAc are 0.2, 0.4, 0.6, 0.8, 1.0, 1.2, 1.4, 1.6 mL. The particle diameter distributions of the corresponding COFs were shown in Figure 2B–G. Respectively, the average particle sizes of the COFs are ~1.1 μm (Figure 2A), ~800 nm (Figure 2B), ~700 nm (Figure 2C), ~400 nm (Figure 2D), ~350 nm (Figure 2E), ~300 nm (Figure 2F), ~270 nm (Figure 2G), ~250 nm (Figure 2H). It can be clearly seen from the figure that with the increase in the amount of HAc, the size of the COF nanomaterials gradually decreased. At the same time, the morphology also changed significantly. The more HAc that was used, the more burrs appeared on the outer layer of COFs, changing from the initial sphere to gradually become more flower-like. As the particle size decreased, the specific surface area increased, and the outer surface became rough. This not only creates a favorable environment for the attachment of AuNPs, but also lays the foundation for the construction of electrochemical sensing experiments based on AuNPs@COF nanocomposites.

**Figure 3:**
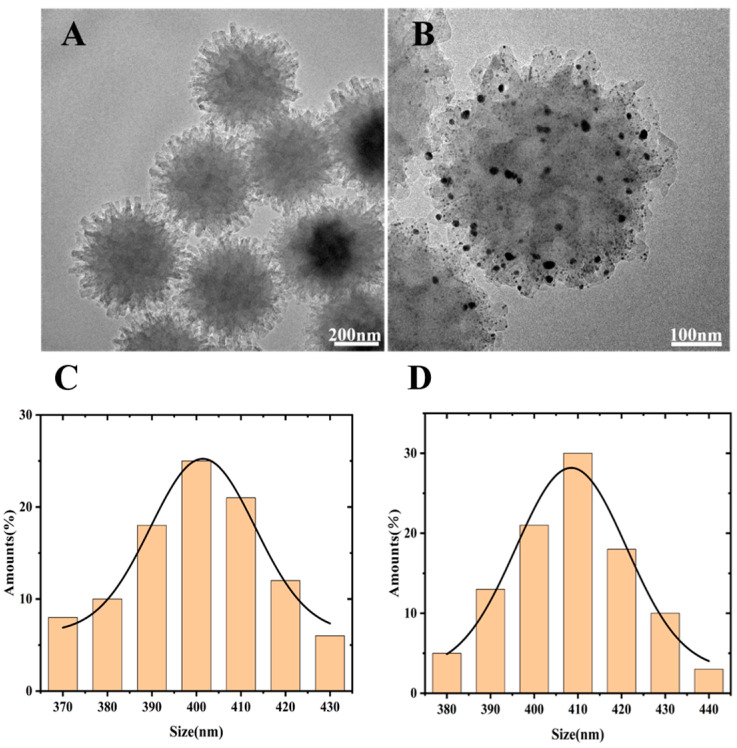
TEM images of COF nanomaterials (**A**) and AuNPs@COF nanocomposites (**B**); COF nanomaterials (**C**); and AuNPs@COFs nanocomposites’ (**D**) particle diameter distribution.

Firstly, the optimized COFs nanomaterials were screened, and then the selected COFs nanomaterials with the best morphology and size were functionalized with AuNPs. The prepared COFs nanomaterials and AuNPs@COFs were characterized by TEM. The results are shown in Figure 3A,B. The COF nanomaterials prepared in Figure 3A are uniform in size and neatly arranged. The selected material was prepared by 0.8 mL HAc with a particle diameter distribution in the range of 370–430 nm and an average particle size of about 400 nm, as shown in Figure 3C. Relatively, the particle diameter distribution range of AuNPs@COFs nanocomposites was 380–440 nm and the average particle size was 410 nm (Figure 3D). The nanostructure is similar to a sea urchin, which is caused by the polycondensation reaction during the catalytic process. This structure is conducive to ion transfer during the electrochemical catalytic process. At the same time, the larger specific surface area of COFs nanomaterials provides more loading sites, and can also prevent AuNPs from agglomeration during growth to some extent. In Figure 3B, the flower-like nanostructure of AuNPs@COF was prepared using the in situ reduction method under the action of sodium citrate. The black small particles attached in Figure 3B are dispersed AuNPs with the particle size of 7 nm. The successful attachment of AuNPs greatly improves the electrical conductivity of the COFs material and allows for a large amount of space inside and outside the structure, which is beneficial to electron transfer and conduction.

**Figure 4:**
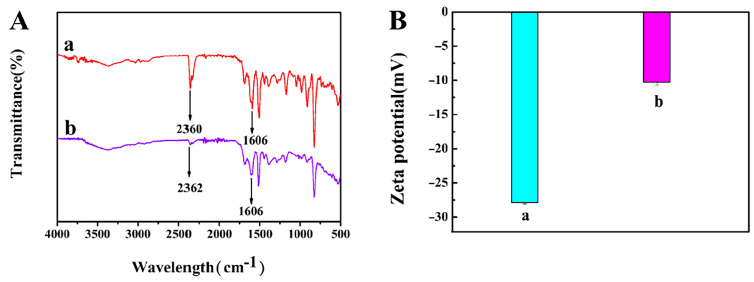
(**A**) FTIR spectra of COFs nanomaterials (a) and AuNPs@COFs nanocomposites (b); (**B**) zeta plots of COFs nanomaterials (a) and AuNPs@COFs nanocomposites (b).

The functional groups formed during the preparation of the material were analyzed by FTIR [28]. Figure 4A (a) shows that a characteristic peak belonging to C=N appears in COFs at 1606 cm^−1^, which indicates that the aldehyde group of DVA and the amino group of TAPB undergo condensation reaction under the action of catalyst. As can be seen from Figure 4A (b), after the introduction of AuNPs on the surface of the COFs, the presence of the characteristic peak C=N at 1606 cm^−1^ is the same as that of COFs, further indicating that the structure of COFs is still intact and not affected by AuNPs doping. In addition, it is worth noting that the observed absorption band shifted from 2360 cm^−1^ to 2363 cm^−1^, which may be attributed to the electrostatic interaction between NH_3_^+^ and [AuCl_4_]^−^ [31]. Thus, the AuNPs@COFs nanocomposites were successfully prepared. Zeta potential analyzer was used to study the charge on the surface of nanomaterials before and after composite. Figure 4B, (a) shows that the charge on the surface of COFs material was −27.86 mV, and (b) shows that the charge on the surface of AuNPs@COFs nanocomposites was −10.28 mV. Due to the positive charge of AuNPs, the negative charge on the surface of the nanocomposites slightly reduced. In summary, the surface of COF nanomaterials was negatively charged before and after functionalization.

### 3.2. Electrochemical Performance of AuNPs@COFs Nanocomposites

**Figure 5:**
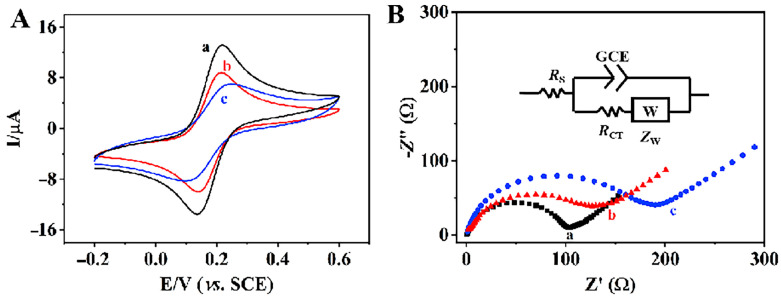
(**A**) CV curves of AuNPs@COFs (a), GCE (b) and COFs (c) in in 0.5 mmol/L [Fe(CN)_6_]^3−/4−^ and 0.1 mol/L KCl electrolyte solution; (**B**) EIS of AuNPs@COFs nanocomposites (a), GCE (b) and COFs (c) in 0.5 mmol/L [Fe(CN)_6_]^3−/4−^ and 0.1 mol/L KCl electrolyte solution.

The electrochemical properties of different modified electrodes in [Fe(CN)_6_]^3−/4−^ solution were studied by cyclic voltammetry (CV). Figure 5A shows the CV curves of different modified electrodes with a pair of well-defined redox peaks, characteristic of the [Fe(CN)_6_]^3−/4−^ redox processes. The redox current of COFs/GCE(c) electrode was lower than that of the bare GCE(b) electrode, which may be due to the poor conductivity of COFs. The redox current of COFs/GCE(c) electrode was much lower than that of the AuNPs@COFs/GCE(a) electrode. The enhancement of redox current of the AuNPs@COFs/GCE(a) electrode may be due to the direct band gap and high electron transfer ability of AuNPs, which brought the AuNPs@COFs excellent conductivity, strong catalytic activity and a large specific surface area. The results show that AuNPs@COFs exhibit the strongest peak current response, indicating that the synergistic effect of the nanocomposites improves the electron transfer rate and catalytic activity. The heterogeneous electron transfer rate (k^0^) was also calculated using the method developed by Klingler and Kochi [32]. The results showed that AuNPs@COFs/GCE exhibited faster transfer kinetics with k^0^ = 2.74 × 10^−3^ cm s^−1^, which was faster than that of COFs/GCE (k^0^ = 1.08 × 10^−3^ cm s^−1^). The results show that AuNPs@COFs have good levels of conductivity and are suitable as electrode modification materials for electrochemical sensors.

In order to study the electrochemical properties of different materials modified electrodes, the interface properties of different electrodes were evaluated by electrochemical impedance spectroscopy (EIS) in the frequency range of 0.01–100 kHz. Figure 5B shows the Nyquist plots of different modified electrodes, where the semicircle diameter in the high frequency region represents the electron transfer resistance and the linear portion in the low frequency region represents the diffusion resistance [33]. Nyquist plots (Figure 5B) consisting of an imaginary part (Z′′) versus a real part (Z′) were simulated according to the Randles equivalent circuit model shown in Figure 5B—inset. The circuit consists of the R_S_ component, which is the ohmic resistance from the solution, in series with the combination of constant phase element (GCE) combined in parallel with the charge transfer resistance (R_CT_) of the Faradaic process and the Wargburg impedance (Z_W_) component arising from the mass diffusion towards the electrode surface. Herein, the GCE component is incorporated instead of the true double layer capacitance (C_dl_) due to heterogeneity in the electrode surface that contributes to the imperfect capacitance. The Rct value of COFs (c) was about 170 Ω and the conductivity was low. The Rct value of the bare GCE (b) was about 130 Ω, whereas the Rct value of AuNPs@COFs (a) decreased to about 90 Ω, which is due to the good conductivity of AuNPs and almost no agglomeration on the surface of COFs. It shows that different modified components have obvious synergistic effects. This result is consistent with the above CV curve. In summary, AuNPs@COF nanocomposites exhibit good charge transfer kinetics and a fast electron transfer.

**Figure 6:**
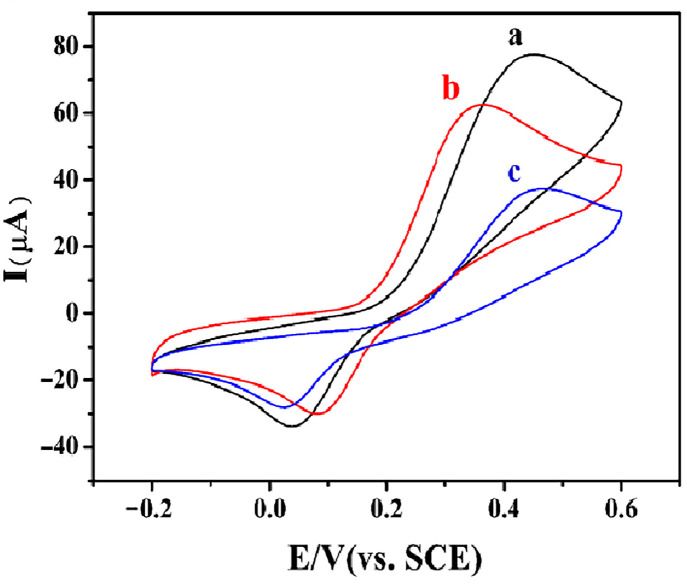
CV curves of AuNPs@COFs (a), GCE (b) and COFs (c) in 0.1 mol/L PBS electrolyte containing 0.6 mmol/L ractopamine.

The response of different modified electrodes to ractopamine was studied by cyclic voltammetry (CV). As shown in Figure 6, the peak current response of COFs/GCE (c) to ractopamine was lower than that of bare GCE (b), which may be due to the poor conductivity of COF, and the introduction of AuNPs significantly improved the peak current response of COFs/GCE. AuNPs@COFs/GCE (a) showed the strongest peak current response to ractopamine, indicating that the composite material improved the electron transfer rate and catalytic activity; therefore, AuNPs@COFs/GCE was selected for ractopamine detection.

**Figure 7:**
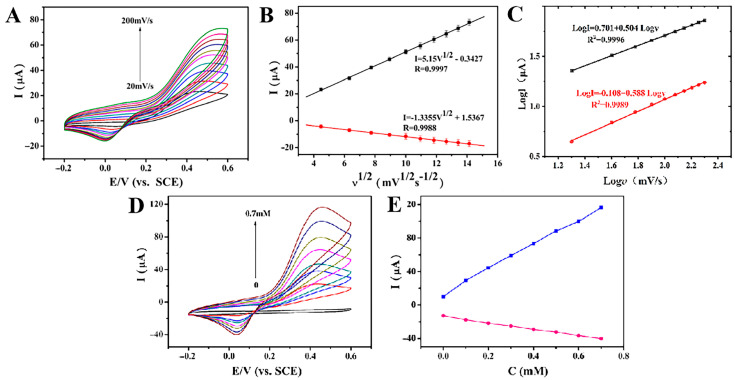
CV curves of AuNPs@COFs nanocomposites to 1 mmol/L ractopamine at different scan rates (20, 40, 60, 80, 100, 120, 140, 160, 180, 200 mV/s) in 0.1 mol/L PBS (**A**) and the relationship between peak current and square root of scan rate (**B**); corresponding logI vs. log*v* plots (**C**); CV curves of AuNPs@COFs nanocomposites for different concentrations of ractopamine (0, 0.1, 0.2, 0.3, 0.4, 0.5, 0.6 and 0.7 mmol/L) (**D**); linear relationship between ractopamine concentration and ractopamine peak current (**E**).

In order to study the electrode reaction kinetics, the effect of scanning rate on ractopamine current and potential was studied using the CV method. In Figure 7A, when the concentration of ractopamine was 1 mmol/L and the scan rate increased from 20 mV/s to 200 mV/s, the redox peak current of ractopamine increased linearly. As shown in Figure 7B, the anode and cathode peak currents of ractopamine are fitted to the square root of the scanning speed (υ^1/2^). The regression equation is as follows:

Ipa = 5.15υ^1/2^(mV/s)^1/2^ − 0.3427 (R^2^ = 0.9997) (1)

Ipc = −1.3355υ^1/2^(mV/s)^1/2^ + 1.5367 (R^2^ = 0.9988) (2)

The oxidation peak current and reduction peak current are proportional to the square root of the scan rate, which is consistent with the quantitative relationship between Ip and υ^1/2^ in the Randles–Sevcik equation, indicating that the electron loss process on the surface of the AuNPs@COFs composites is controlled by diffusion [34]. It shows that the material has high catalytic performance under ractopamine. Moreover, with the increase in the scanning rate, the anodic peak potential (Epa) of ractopamine shifts positively and the cathodic peak potential (Epc) shifts negatively. This is due to the kinetic control of the redox reaction process. The relationship curves between logI and logυ were also studied. From Figure 7C, it could be seen that the slopes of Figure 7B curves corresponding to logI and logυ were 0.504 and 0.588, respectively, reflecting the fast kinetics dominated by pseudocapacitance process.

In order to further investigate the electrochemical catalytic activity of AuNPs@COFs nanocomposites for ractopamine, CV analysis was performed at a fixed scanning rate (100 mV/s) and a certain ractopamine concentration range (0, 0.1, 0.2, 0.3, 0.4, 0.5, 0.6, 0.7 mmol/L) (Figure 7C). The results showed that with the increase in ractopamine concentration, the oxidation peak current of ractopamine gradually increased and the amplitude was larger, whereas the reduction peak current increased less, indicating that the oxidation peak current was more sensitive to the determination of ractopamine. Figure 7D,E show that the concentration of ractopamine is positively correlated with the oxidation peak and reduction peak current of ractopamine.

### 3.3. Amperometric Detection of Ractopamine

**Figure 8:**
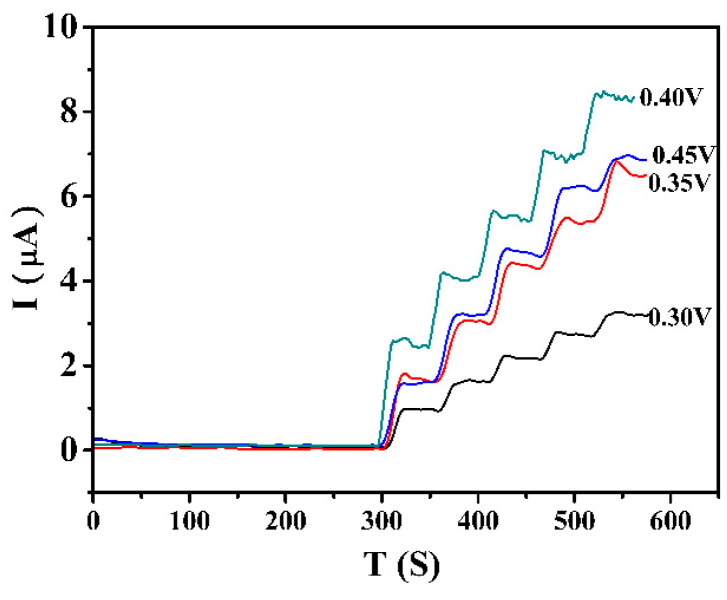
I-T plots of AuNPs@COFs/GCE to ractopamine at different potentials in 0.1 mol/L PBS solution.

It is necessary to optimize the working potential before quantitative analysis of ractopamine. The working potential affects the sensitivity of the sensor. Figure 8 is the I-T diagram of AuNPs@COFs nanocomposites measured under the condition of changing the concentration of ractopamine (adding 10 μL 0.1 mol/L ractopamine every 50 s) by continuously and stably stirring after changing different working potentials. It was found that the current reached the maximum voltage at 0.40 V when the working potential increased from 0.30 V to 0.45 V. Therefore, 0.40 V was selected as the optimal potential and used for subsequent quantitative analysis.

**Figure 9:**
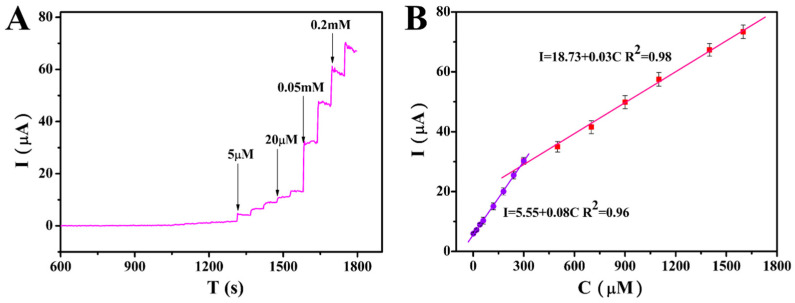
(**A**) The I-T diagram of AuNPs@COFs/GCE for ractopamine was obtained by adding different concentrations of ractopamine into 0.1 mol/L PBS solution at 0.40 V applied voltage. (**B**) Linear fitting curve of ractopamine concentration and current value.

According to the above method, ractopamine was added to 10 mL PBS electrolyte every 50 s. Figure 9A shows the process of current changing with time. With the continuous addition of ractopamine, the current showed a stepwise upward trend. There are two corresponding current and concentration calibration diagrams for the oxidation of ractopamine by AuNPs@COFs/GCE in different ractopamine concentration ranges. The linear relationship is I (μA) = 0.08C (μmol/L) + 5.55 in the range of 1.2–280 μmol/L, and I (μA) = 0.03C (μmol/L) + 18.73 in the range of 280–1600 μmol/L (Figure 9B). R^2^ is 0.96 and 0.98, respectively. When the ractopamine concentration was high (>3.1 mmol/L), the current was nonlinear, which may be ascribed to the saturation of the catalytic sites on the working electrode surface. The calculated LOD [35] is 0.12 μmol/L. In order to confirm the accuracy and precision of this method, we also performed F test and *t* test. The t values of the two linear ranges were 12.81 and 14.53, and the F values were 1918.98 and 932.27, *p* < 0.01, respectively, which shows that the method has a significant linear relationship, and good accuracy and precision. Compared with other reported ractopamine sensors (Table 1), AuNPs@COFs/GCE has a wider linear range, which indicates that AuNPs@COFs nanocomposites are good catalysts for ractopamine.

### 3.4. Selectivity, Stability and Repeatability Analysis of Proposed Sensor

**Figure 10:**
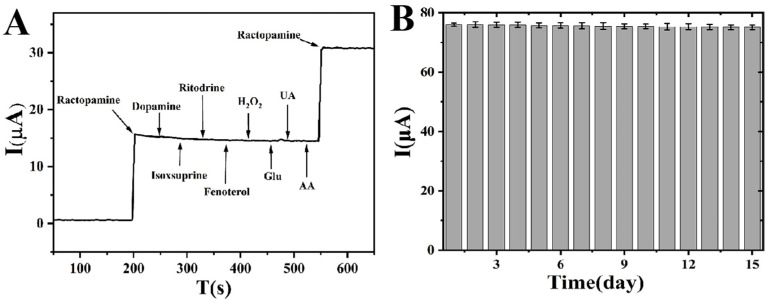
I-T diagram (**A**) in response of AuNPs@COFs electrode in 0.1 mol/L PBS solution when ractopamine, dopamine, isoxsuprine, ritodrine, fenoterol, H_2_O_2_, Glu, UA, AA and ractopamine were added in turn (the concentration of ractopamine added was 0.3 mmol/L, and the concentration of interfering substances added was 3 mmol/L); stability of the electrochemical sensor based on AuNPs@COF nanocomposites (**B**).

Anti-interference ability is one of the most important indexes to test the practicability of electrode. AuNPs@COFs nanocomposites were used to detect some electroactive substances coexisting with glucose in real samples. Potential interferences such as hydrogen peroxide (H_2_O_2_), ascorbic acid (AA), glucose (Glu), uric acid (UA), dopamine, isoxsuprine, ritodrine, and fenoterol were detected individually using amperometric assay. As shown in Figure 10A, when 3 mmol/L dopamine, isoxsuprine, 3 mmol/L ritodrine, 3 mmol/L fenoterol, 3 mmol/L Glu, 3 mmol/L H_2_O_2_, 3 mmol/L UA, and 3 mmol/L AA were added to the system in turn, the response current value remained basically unchanged. However, significant responses were obtained after adding ractopamine. These indicate that AuNPs@COFs/GCE has good anti-interference ability with ractopamine. In addition, six AuNPs@COFs/GCE electrodes were prepared using the same method to test the reproducibility. The relative standard deviation (RSD) was 0.51%, and the maximum increase of the current response was 5.31%, indicating that AuNPs@COFs/GCE have good reproducibility. It can be seen from Figure 10B that the prepared electrode was stored at 4 °C for 15 days to test its long-term stability. The results show that the response current maintained almost 96.4% of its original current, indicating that the sensor based on AuNPs@COF nanocomposites has good stability.

### 3.5. Detection in Actual Samples

The applicability of electrochemical sensors based on AuNPs@COFs nanocomposites was evaluated. Pork and chicken were selected for further detection in this study. The ractopamine in actual samples was analyzed by standard addition method and compared with HPLC. It is found that the results of HPLC and AuNPs@COFs/GCE for the detection of ractopamine content in meat were basically the same (Table 2). The recovery rate of ractopamine by the sensor was 95.0–108.1% and RSD was 2.13–5.12%. All actual sample detection was performed in meat extracts. This indicates that the sensor can be applied to the detection of ractopamine in actual samples.

## 4. Conclusions

In summary, COFs have potential expansion value in the field of food safety detection due to their simple synthesis method, stable structure and environmental friendliness. Metal nanoparticles, especially AuNPs, as an efficient catalyst, promote electron transfer on the AuNPs@COF-based electrochemical sensor, overcoming the defects of single metal nanoparticles while combining the advantages of magnetic nanomaterials. In this paper, COFs nanomaterials were synthesized by polycondensation reaction at room temperature, and then AuNPs@COF nanocomposites were prepared by modifying AuNPs on the surface. Subsequently, the nanocomposites were modified on the electrode surface to successfully construct a AuNPs@COFs/chitosan/GCE for ractopamine detection. The results showed that the linear range of ractopamine detection was 1.2–1600 μmol/L and the limit of detection (LOD) was 0.12 μmol/L. Compared with the same type of ractopamine sensor, the sensor based on AuNPs@COF nanocomposites has a wider linear range, lower detection limit, higher stability and better selectivity. The electrochemical sensing platform has been successfully applied to the determination of ractopamine in meat extracts with good accuracy and reliability. Food safety detection methods based on functional nanomaterials will usher in new development opportunities in future.

## Figures and Tables

**Table 1:** Performance comparison of different modified electrodes for ractopamine electrocatalytic oxidation.

Electrode *	Linear Range (μmol/L)	Limit of Detection (LOD) (μmol/L)	Reference
SMWCNT-NF/ITO	0.3–33.3	0.1	[35]
PoAT-AuNP-Au/SCE	2.5–150	1.17	[36]
FLG-PI/FGE	5–80	1.29	[37]
	10–200	2.7	
	25–250	7.81	
NiFe_2_O_4_/GCE	2–60	1	[38]
Nafion/HPMo/MoS_2_/PDDA/GCE	1–70	0.06	[39]
AuNPs@COFs/GCE	1.20–280280–1600	0.12	This work

* SMWCNT-NF: Single-walled carbon nanotubes-nafion; PoAT: Poly(o-aminothiophenol); FLG-PI: Layers of graphene-polyimide film; HPMo: Polyoxometalate H_3_PMo_12_O_40_. PDDA: Polydiene dimethyl ammonium chloride solution.

**Table 2:** Recovery and relative standard deviation (RSD, %, *n* = 3) of ractopamine in meat by electrochemical sensor and HPLC, respectively.

Sample	Ractopamine Added (mmol/L)	HPLC Determination Concentration (mmol/L)	Electrochemical Sensor		
			Determination Concentration (mmol/L)	Relative Standard Deviation (RSD)	Recovery Rate
Sample 1	0.05	0.08	0.08	2.51%	98.0%
	1.00	1.06	0.94	5.12%	95.2%
	2.00	2.10	2.08	3.13%	101.0%
Sample 2	0.1	0.16	0.15	4.01%	97.5%
	1.00	1.10	1.07	3.73%	98.3%
	2.00	2.09	2.11	2.13%	108.1%

## Data Availability

The data presented in this study are available on request from the corresponding author.
